# Development and Characterization of a Novel Fusion Protein of a Mutated Granulocyte Colony-Stimulating Factor and Human Serum Albumin in *Pichia pastoris*

**DOI:** 10.1371/journal.pone.0115840

**Published:** 2014-12-23

**Authors:** Yan-Shan Huang, Xiao-Fang Wen, Zhi-Yu Yang, Yi-Liang Wu, You Lu, Lin-Fu Zhou

**Affiliations:** 1 Medical Biotechnology Laboratory, Zhejiang University, Hangzhou, China; 2 Jiangsu T-Mab Biopharmaceutical Co., Ltd., Jiangsu, China; Aligarh Muslim University, India

## Abstract

The purpose of the present work was to develop a novel, long-acting and potent human serum albumin/granulocyte colony stimulating factor (HSA/G-CSF) therapeutic fusion protein. The novel fusion protein, called HMG, was constructed by genetically fusing mutated human derived G-CSF (mG-CSF) to the C-terminal of HSA and then prepared in *Pichia pastoris*. The molecular mass of HMG was about 85 kDa and the isoelectric point was 5.3. Circular dichroism spectroscopy suggested that mG-CSF retained nearly all of its native secondary structure, regardless of fusion. The binding capabilities of mG-CSF moiety to G-CSF receptor and HSA moiety to warfarin showed very little change after fusing. The bioactivity of HMG (11.0×10^6^ IU/mg) was more than twice that of rHSA/G-CSF (4.6×10^6^ IU/mg). A mutation was made at the 718^th^ amino acid of HMG, substituting Ala for Thr, to investigate the glycosylation of HMG expressed in *P. pastoris*. Data indicated that HMG was modified at Thr718, speculatively with the addition of a mannose chain. In conclusion, a novel HSA/G-CSF fusion protein was successfully constructed based on a mutated G-CSF. This protein showed more potent bioactivity than rHSA/G-CSF and thus may be a suitable long-acting G-CSF.

## Introduction

For therapeutic proteins and peptides that can be cleared from the body quickly by renal filtration, albumin fusion technology is an attractive means of increasing their circulation time *in vivo*. HSA (human serum albumin) has a molecular weight of 66.5 kDa and exhibits a long half-life of about 19 days *in vivo* [1], [2]. Fusion with HSA increases the molecular weight of proteins and peptides and so prolongs their half-lives *in vivo*. In addition, the albumin molecule masks the protein, which renders it more resistant to proteases and less immunogenic. Albiglutide, a GLP-1/albumin fusion protein, is the first albumin fusion based long-acting drug. It received EMEA and FDA approval in early 2014. The approval of Albiglutide may usher in a promising future for the use of albumin fusion technology as a platform for drug half-life extension. Unfortunately, decreases in the bioactivity of therapeutic proteins and peptides after fusion with HSA are frequent due to steric hindrance. Fleer *et al*. reported that Neugranin, a recombinant human G-CSF (rhG-CSF) genetically fused to human albumin, only retained 14.3% of its bioactivity [3]. In this way, the clinical dosage of Neugranin was increased to 9 mg/cycle (calculated using the G-CSF motif) in contrast to 1–2 mg/cycle of rhG-CSF. Drastically reduced potency causes an intense need for larger clinical doses and increases costs and immunogenicity. This is a major obstacle for the development and application of fusion technology. One common way of resolving this problem is to optimize their linker peptide [4]–[6]. However, little improvement was observed when a linker peptide like GGGGS was inserted.

For this reason, a different strategy was designed in current study to generate a novel albumin/G-CSF fusion protein based on a highly potent G-CSF mutant. Thr-1, Leu-3, Gly-4, Pro-5, and Cys-17 in the G-CSF mutant were each substituted with Ala, Thr, Tyr, Arg, and Ser, respectively and this G-CSF mutant showed more potent granulopoietic activity than the intact rhG-CSF both *in vitro* and *in vivo* [7]. This novel fusion protein, called HMG, was expressed in *P. pastoris* and the structural integrity, conformational stability, ligand binding profile, and bioactivity of HMG and its two components (mG-CSF and HSA) were examined. Additionally, we designed a mutation in the HMG (called mHMG, substituting Ala for Thr at 718 position of HMG) to explore the glycosylation site and status of HMG.

## Materials and Methods

### Construction, expression, and purification of recombinant proteins

Human G-CSF mutant (mG-CSF) was prepared as previously described [7]. Recombinant HMG was constructed by genetically fusing mG-CSF to the C-terminus of HSA. mHMG, a mutated HMG, was achieved by replacing Thr with Ala at 718 position of HMG. Then, HMG, mHMG, and rHSA/G-CSF were cloned into the eukaryotic expression vector pPIC9 and expressed in *P. pastoris* as previously described [8]. The original α factor signal peptide in pPIC9 (Invitrogen, USA) was replaced with native HSA signal peptide to direct the secretion of nascent proteins. mG-CSF was cloned into a prokaryotic expression vector pET39b(+) using the restriction enzyme *Nde*I and *Eco*RI and prepared as described by Kuga *et al.* [7]. All protocols and fermentation procedures were conducted according to the manufacturer's instructions. Briefly, after 2 days of culture at 30°C using a Biostat C 15 L fermenter (B.Braun, Germany), *P. pastoris* were stimulated by continuous addition of methanol for about 50 h to induce the expression of proteins. Recombinant proteins were collected from the fermentation broth by centrifugation (5000 rpm). Purification of HMG, rHSA/G-CSF, and mHMG was performed as follows: Cibacron Blue sepharose FF chromatography, phenyl sepharose HP chromatography, Sephadex G25 for buffer exchange, SP sepharose FF chromatography, and a final ultrafiltration/diafiltration (30K MWCO). Purification of mG-CSF was accomplished as follows: DEAE sepharose FF chromatography, phenyl sepharose HP chromatography followed by a final ultrafiltration/diafiltration (10K MWCO). Purified proteins were stored frozen in 5 mg/ml buffer consisting of 20 mM sodium phosphate, pH 7.3.

### SDS-PAGE, isoelectric focusing electrophoresis (IEF), and size exclusion chromatography (SEC-HPLC) analysis

The fermentation solution and purified HMG fusion proteins from different purification processes were analyzed using sodium dodecyl sulfate–polyacrylamide gel electrophoresis (SDS–PAGE) with 8% acrylamide gel and 5% condensing gel in the Mini-Protein II electrophoresis unit (Bio-Rad, USA) and stained with 0.25% Coomassie brilliant blue R-250 (Aldrich, USA).

IEF was used to predict the isoelectric point (pI) of HMG. In a separate set of experiments, 2 µg of purified HMG, mG-CSF, HSA and a mixture of HSA, mG-CSF and HMG prepared in 20 mM PB (phosphate buffer, pH 7.4) were loaded and analyzed on a Pharmacia MultiphorII horizontal electrophoresis system (GE Healthcare, USA) using ampholine, pH 3.5–10 (GE Healthcare, USA).

These samples were also analyzed using size exclusion chromatography on a TSK-GEL G3000SW columns (7.5×300 mm) (Tosoh, Japan) at a flow rate of 0.6 ml/min in 20 mM sodium phosphate (pH 7.5) and 0.15 M NaCl. The absorbance was monitored at 280 nm.

### N- and C-terminal amino acid sequencing

N-terminal amino acid sequencing was performed by Edman degradation with Shimadzu PPSQ-33A automated protein sequencer. C-terminal amino acid sequencing was performed with Micromass QTOF2 Quadrupole/Time-of-Flight Electrospray ionization tandem mass spectrometry (Q-TOF2 ESI-MS/MS).

### Circular dichroism (CD) spectroscopy

Far and near-UV CD spectra of equimolar mixtures of HSA and mG-CSF (abbreviated as emHmG, 0.5 mg/ml, in 20 mM PB, pH 7.4) and HMG fusion protein (0.5 mg/ml, in 20 mM PB, pH 7.4) were recorded on a JASCO J-715 automatic recording spectropolarimeter (JASCO, Japan) from 190–250 nm and 250–300 nm, respectively.

### Intrinsic fluorescence measurements

Intrinsic fluorescence emission spectra were used to detect possible conformational changes in mG-CSF after fusion with HSA. mG-CSF, HSA, and HMG were prepared using 67 mM phosphate buffer (PB, pH 7.4) as follows: a: mG-CSF (15 µM), b: HSA (15 µM) plus mG-CSF (15 µM), and c: HMG (15 µM). The samples (200 µl/well) were pipetted into a 96-well black plate (Costar, USA) and PB was added as a negative control. The plate was placed in SpectraMax M5 (Molecular Devices, USA) to examine the changes in intrinsic fluorescence under 25°C. The excitation wavelength was set to 295 nm and emission wavelength was from 320 to 380 nm.

### Warfarin binding properties of HMG and HSA

The interaction of warfarin and HSA was examined using fluorescence spectroscopy. Human-plasma-derived HSA (Octapharma, Austria), HMG and warfarin sodium (Adamas-Beta, China) were prepared with 0.01 M phosphate buffered saline (pH 7.4) as follows: a: 5 µM HSA, b: 5 µM HSA plus 50 µM warfarin sodium, c: 5 µM HMG and d: 5 µM HMG plus 50 µM warfarin sodium. The samples were then pipetted into a 96-well black plate (Costar, 200 µl/well) and detected using a microplate reader (SpectraMax M5, Molecular Device, USA). The excitation wavelength was set to 320 nm and fluorescence intensity was monitored at 380 nm. The experiment was independently repeated three times.

### G-CSF receptor (G-CSFR) binding assay of HMG, rhG-CSF, and mG-CSF

Bio-layer interferometry (BLI) was used to detect the binding of G-CSF to G-CSFR under various conditions using a Streptavidin High Binding Biosensor Kit and a Octet-QK system (Fortebio, USA). Biotinylated rhG-CSFR was desalted with Sephadex G25 (GE Healthcare, USA) and eluted in a final concentration of 15 µg/ml. Sample preparation, hydration of the sensors, and kinetic analysis of macromolecular interactions were performed according to the manufacturer's instructions. Then 200 µl of HMG, rhG-CSF standard (Amgen, USA), and mG-CSF or buffer were transferred into a 96-well plate (Greiner, Austria) and assayed for rapid assessment of kinetics. Data were analyzed using global fitting to determine the association/dissociation rate constants and affinity constant.

### Glycosylation modification analysis using periodic acid-Schiff staining, ConA chromatography, and phenol-sulfuric acid reaction

Periodic acid–Schiff (PAS) staining is used to detect sugar compounds such as polysaccharides, glycoproteins, and glycolipids. The reaction of periodic acid oxidizes the vicinal diols in glycosyl and creates a pair of aldehydes at the two free tips of each broken monosaccharide ring. These aldehydes then react with the Schiff reagent and form purple-magenta compounds. Purified protein samples (5 µg and 20 µg), a positive control (horseradish peroxidase), and a negative control (soybean trypsin inhibitor) were loaded on SDS-PAGE, subjected to electrophoresis, and then separately stained with periodic acid-Schiff reagent (Thermo Scientific, USA) and Coomassie blue R-250.

The carbohydrate content of the three batches of purified HMG or emHmG was further determined by phenol-sulfuric acid method based on Dubois *et al.* [9]. A series of sucrose solutions with different concentrations, 0 µg/ml, 10 µg/ml, 20 µg/ml, 30 µg/ml, 40 µg/ml, 50 µg/ml, and 80 µg/ml were prepared in tubes by diluting 1% sucrose stock solution (containing 0.5% concentrated sulfuric acid V/V) with deionized water. Then 1 ml of 9% phenol solution was added to the tubes and mixed well, followed by quick addition (within 5–20 s) of 5 ml concentrated sulfuric acid. This mixture was then mixed well and transferred to a boiling water bath for 30 min. Each solution was chilled to ambient temperature in ice water and then absorbance was recorded at 490 nm and plotted against the sucrose concentrations.

Concanavalin A (ConA) affinity chromatography was conducted to further characterize the glycosylation modification of HMG. ConA, a lectin carbohydrate-binding protein originally extracted from jack-bean (*Canavalia ensiformis*), can specifically bind to certain structures found in various sugars, glycoproteins, and glycolipids, mainly internal and nonreducing terminal α-D-mannosyl and α-D-glucosyl groups. Because of this, it can be used to separate proteins containing certain sugars from those that do not contain them. In a separate experiment, 30 mg quantities of purified HMG fraction and mHMG were prepared in 0.5 mol/l NaCl and then subjected to affinity chromatography with a ConA Sepharose 4B column (GE Healthcare, USA) which had been previously equilibrated with 0.5 M NaCl in 20 mM Tris-HCl (pH 7.2). Finally, the proteins were eluted using 0.2 mol/l glucoside.

### Mass spectrometric analysis

An Agilent 6224 Accurate-Mass Time-of-Flight (TOF) LC/MS system equipped with an Agilent 1260 Infinity LC and electrospray ionization (ESI) was used to investigate the possibility of glycosylated isomers and other posttranslational modifications that might occur in HMG. LC column is packed with polymer-based matrix (polystyrene divinylbenzene). The mobile phase was a: 0.1% formic acid in Milli-Q water and b: 0.1% formic acid in acetonitrile. For ESI-TOF, ion spray voltage and gas temperature were set to 3500 V and 325°C, respectively.

### *In vitro* bioactivity assay

Bioactivity of the fusion protein was determined using a cell proliferation assay using murine myeloblastic NFS-60 cells. Briefly, the cells were seeded in a 96-well plate and incubated in 50 µl of assay medium containing serial dilutions of either HMG or 0.7×10^8^ IU/mg of recombinant hG-CSF standard (Amgen, USA). The cultures were kept at 37°C in a humidified incubator with 5% CO_2_ for two days and then 10 µl of 2.5 mg/ml MTT was added. Four hours later, 100 µl of 10% SDS in 0.01 M HCl was added to lyse the cells and dissolve the formazan. The plate was then read at 570 nm with a reference wavelength of 630 nm. The reading at OD_570_ was plotted against the protein concentrations and the concentration at the inflection point of the sigmoidal curve represented the median effective dose (ED_50_).

## Results

### Expression and purification of HMG and mHMG

Protein expression was induced for 50 h using methanol. Concentrations of HMG and mHMG in broth were then about 0.65 g/l and 0.5 g/l respectively, as detected by SDS-PAGE (data not shown). Blue Sepharose Fast Flow was used to capture protein from broth because the albumins in fusion proteins were able to bind to Cibacron Blue specifically. About 204 mg HMG and 142 mg mHMG were purified from 1 L of cell-free broth, equivalent to total protein recovery rates of 31.3% and 28.4%, respectively.

### Characterization of purified HMG

**Figure 1:**
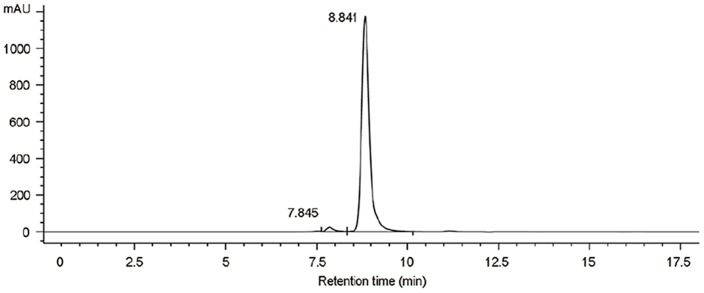
Size exclusion chromatography (SEC) analysis of HMG. Size exclusion chromatography was used to explore the purity of HMG (the fusion protein of HSA and mutated G-CSF).

**Figure 2:**
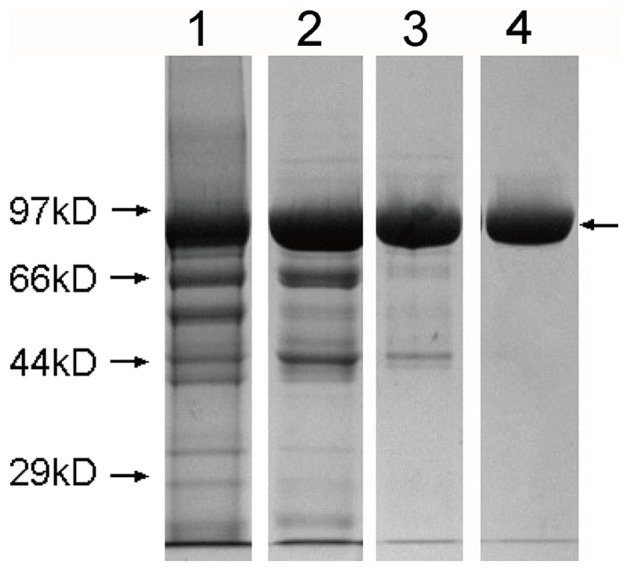
Sodium dodecyl sulphate-polyacrylamide gel electrophoresis (SDS-PAGE) analysis of purified HMG. Lanes 1–4: broth supernatant, fraction of Blue Sepharose FF, fraction of Phenyl Sepharose FF, fraction of Q Sepharose FF. The arrow on the right indicates the band of HMG.

**Figure 3:**
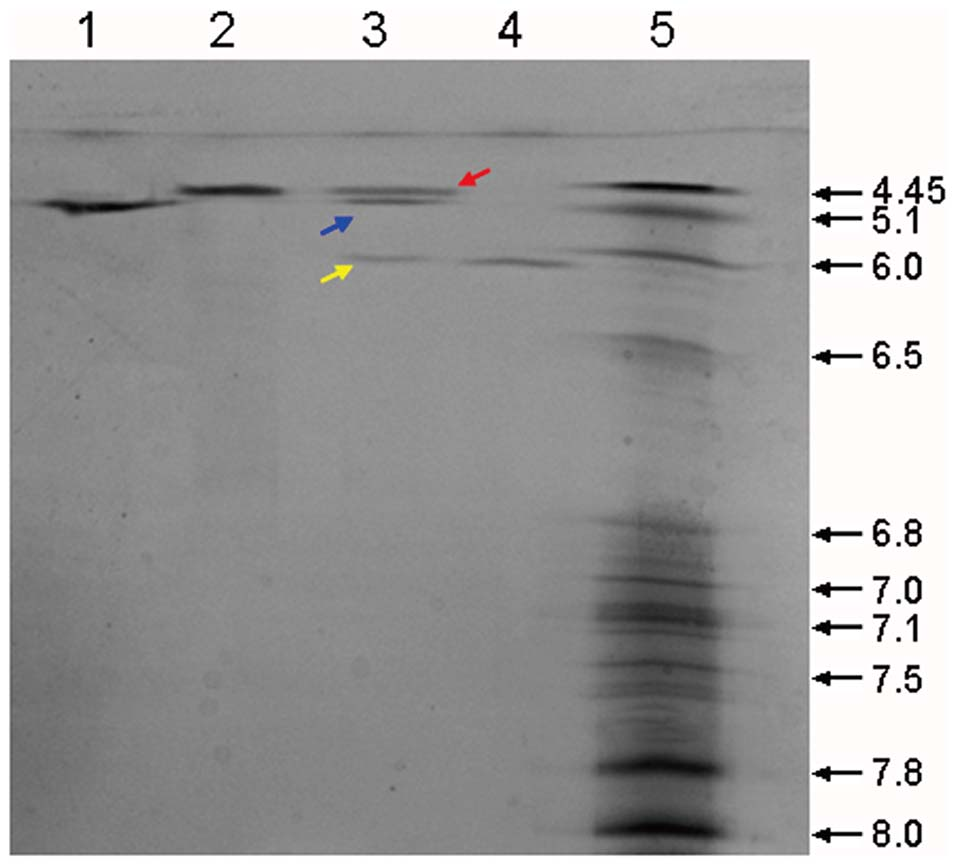
Isoelectric focusing electrophoresis analysis of HMG. Isoelectric focusing electrophoresis was conducted for HMG (Lane 1), HSA (Lane 2), HSA plus mG-CSF and HMG (Lane 3), and mG-CSF (Lane 4). An IEF marker was loaded in Lane 5. The red arrow represents the band of HSA, the blue arrow represents the band of HMG and the yellow arrow represents the band of mG-CSF.

HMG was analyzed after purification using SEC-HPLC and SDS-PAGE. Results indicated purity greater than 97% and molecular mass of about 85 kDa (Fig. 1 and Fig. 2). Only few aggregates were observed. As shown in Fig. 3, HMG showed a single band on the IEF gel and the three batches of product all showed a coincident pI of 5.3, in close proximity to the theoretical value (pI 5.6) calculated from the amino acid composition, which indicated that HMG was charged homogeneously.

N-terminal sequencing showed the following results: NH2-DAHKSEV AHRFKDLG, which is identical to the N-terminal sequence of HSA. The three amino acids at the C-terminal were SKE-COOH, also identical to the predicted sequence.

**Figure 4:**
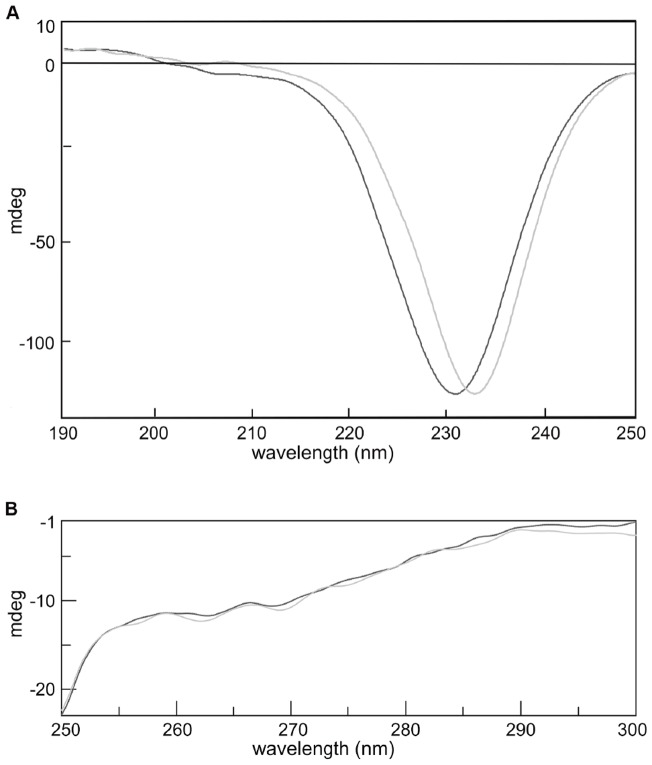
Profiles of circular dichroism spectrum of HMG and emHmG. (A) Far-UV circular dichroism and (B) near-UV circular dichroism. The light gray line represents HMG and the dark gray line represents emHmG. emHmG, equimolar mixture of HSA plus mutated G-CSF

CD spectroscopy was used to analyze the changes in the secondary structure that might occur in mG-CSF after fusion with HSA. Near- and far-UV CD spectra of HMG and emHmG are shown in Fig. 4. Similar CD patterns were observed between HMG and emHmG, indicating that there was little variation in secondary structure after protein fusion.

**Figure 5:**
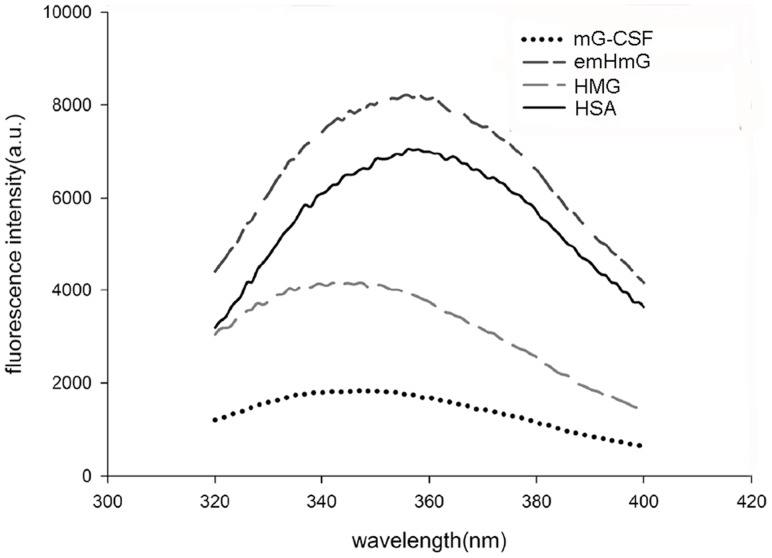
Isoelectric focusing electrophoresis (IEF) profile of HMG. The intrinsic fluorescence emission spectra of HMG, emHmG, mG-CSF, and HSA were measured and used to detect the possible conformational changes in mG-CSF after fusion with HSA.

Three aromatic amino acid residues (tryptophan, tyrosine, and phenylalanine) show contributions to the intrinsic fluorescence of the protein. Intrinsic fluorescence is associated with the tertiary structure of each protein and individual peptides and each one exhibits a characteristic and specific emission profile. There are three tryptophans in the peptide chain of HMG (one in mG-CSF and two in HSA). Possible conformational changes in mG-CSF after fusion with HSA were assessed by measuring the intrinsic fluorescence emission spectra of HMG, emHmG, mG-CSF, and HSA. As illustrated in Fig. 5, mG-CSF showed the least fluorescence intensity. This may be attributed to the deeply buried Trp in its three-dimensional conformation. The patterns of emHmG and HSA were similar, and HMG emitted a specific pattern distinct from emHmG, mG-CSF, and HSA.

### Warfarin binding assay of HMG

HSA binds to a large number of endogenous and exogenous ligands (e.g., drugs), affecting their pharmacokinetics, i.e., rate of distribution and elimination to the tissues [10]. Warfarin produced significantly more intrinsic fluorescence when bound to HSA [11]. Fluorescence spectroscopy is widely used to examine warfarin/HSA interactions. In the present study, this technique was used to investigate the warfarin binding properties of HSA after fusion with mG-CSF. As shown in Table 1, similar fluorescence enhancement at 380 nm was found between HSA and HMG when warfarin was added, suggesting that fusion with mG-CSF did not interfere with the ability of HSA to bind to warfarin.

**Table 1:** Quantitative fluorescence of warfarin-HSA binding interaction.

		Fluorescence at 380 nm ^a^			
		1	2	3	Average
HSA	WF (−)^b^	932.77	960.88	844	912.55
	WF (+)	3063.67	3188.46	3124.99	3125.71
HMG	WF (−)^b^	900.54	949.21	934.63	928.13
	WF (+)	2913.26	3246.79	3086	3082.01
WF (+)		65.52	81.42	80.26	73.47

a: Each experiment was performed in triplicate.

b: Warfarin was not added.

### G-CSF receptor (G-CSFR) binding assay of HMG

The kinetics of the binding interaction of G-CSF and G-CSFR was analyzed by using biolayer interferometry. As illustrated in Table 2, HMG, mG-CSF, and rhG-CSF show affinity (Kd/Ka) rates similar to those of G-CSFR, suggesting that the binding of mG-CSF to its receptor may not be affected by fusion to HSA, but the association and dissociation rate constants of HMG seem to be slightly lower than those of mG-CSF and rhG-CSF.

**Table 2:** Kinetic analysis of G-CSFR binding.

	K_D_ (M)^c^	K_a_ (1/Ms)^a^	K_d_ (1/s)^b^	Full X^2^	Full R^2^
HMG	1.39E-09	2.38E+04	3.32E-05	0.616134	0.990309
mG-CSF	1.20E-09	1.62E+04	1.94E-05	0.611018	0.994991
rhG-CSF	1.97E-09	1.14E+04	2.24E-05	0.59577	0.979051

a: The association rate constant (*K*a) is here defined as the rate of complex formation per second in a 1 M solution of two reaction partners.

b: The dissociation rate constant (*K*d) indicates the stability of this complex.

c: The affinity constant *K*_D_ was calculated using the ratio of *K*d to *K*a.

### *In vitro* bioactivity assay

Regarding bioactivity, a cell proliferation assay revealed that the activity of mG-CSF (205.0×10^6^ IU/mg) was greater than that of rhG-CSF (80.2×10^6^ IU/mg) *in vitro* (Table 3). As expected, a drastic decrease was detected in the activity of mG-CSF after fusion with HSA (11.0×10^6^ IU/mg). The activity of HMG was more than twice that of rHSA/G-CSF (4.6×10^6^ IU/mg). These data indicated that the novel fusion protein, in which a mutated mG-CSF was genetically fused to HSA, was more potently bioactive than rHSA/G-CSF and thus may be a suitable therapeutic agent.

**Table 3:** *In vitro* bioactivity of HMG.

Proteins	Specific bioactivity (IU/mg)	Relative bioactivity (100%)
rhG-CSF	80.2×10^6^	100
mG-CSF	205.0×10^6^	255.6
HMG	11.0×10^6^	13.7
rHSA/G-CSF	4.6×10^6^	5.7

### Glycosylation modification analysis

**Figure 6:**
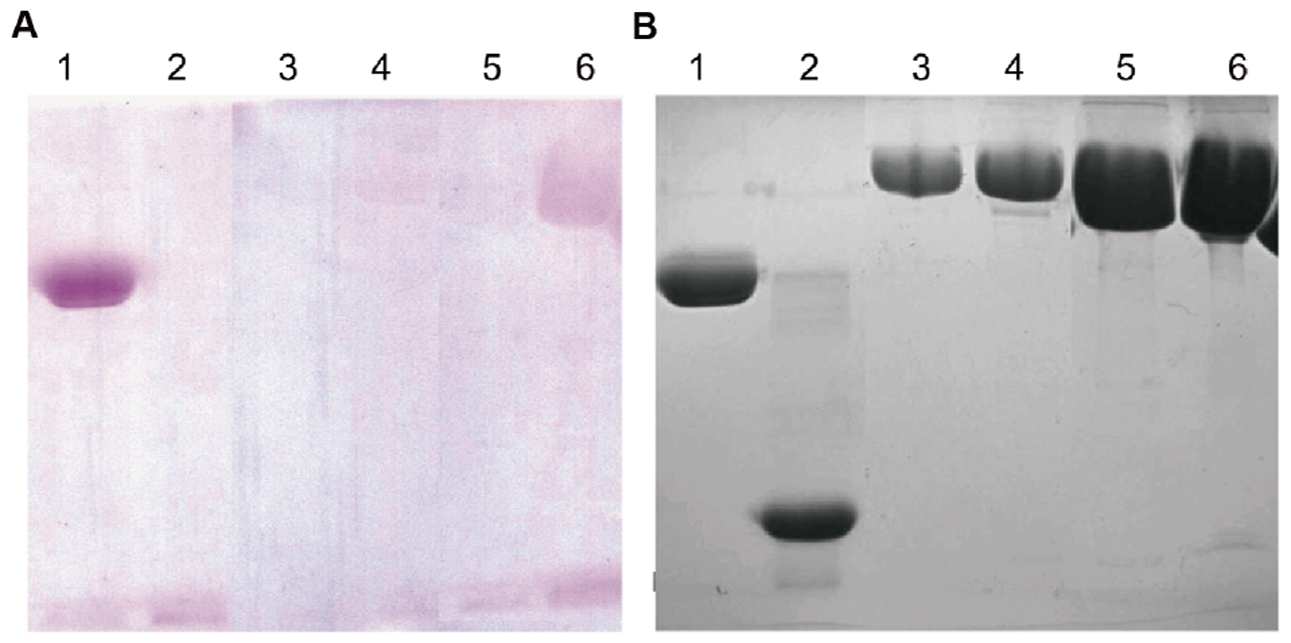
Periodic acid–Schiff and R-250 staining of HMG. A site-directed mutagenesis was made in HMG at position 718 (T718→A718, mHMG) and (A) periodic acid–Schiff staining was performed to detect the galactosylated modifications of HMG. (B) R-250 staining. Lane 1: positive control (horseradish peroxidase); Lane 2: negative control (soybean trypsin inhibitor); Lanes 3 and 5: 5 µg and 20 µg of mHMG, respectively; Lanes 4 and 6: 5 µg and 20 µg of HMG, respectively. mHMG, mutated HMG that T718 in the mG-CSF moiety was mutated to A718.

There is a threonine in the mG-CSF sequence that may possibly be subjected to O-linked glycosylation [12], [13]. Site-directed mutagenesis was performed in HMG at position 718 (T718→A718, mHMG) to explore the potential glycosylation site. Then 20 µg HMG produced a weak purple-magenta band through PAS staining as previously reported [14]. However, 5 µg of HMG did not produce obvious color staining. In contrast, no purple-magenta band was observed when either 5 µg or 20 µg mHMG was loaded (Fig. 6). This indicated that HMG may be slightly glycosylated and that T718 may be a site of O-linked glycosylation.

ConA affinity chromatography showed that HMG bound to ConA strongly, but the vast majority of mHMG flowed through the chromatographic column. This confirmed that glycosylation occurred in HMG.

The phenol-sulfuric acid method was used to quantify glycan content in HMG. Data showed a carbohydrate content approximately of 2.1 µg/mg, equivalent to 1.1 mannose residue per HMG molecule. This was calculated based on the MW (molecular weight) of HMGand mannose residue.

**Figure 7:**
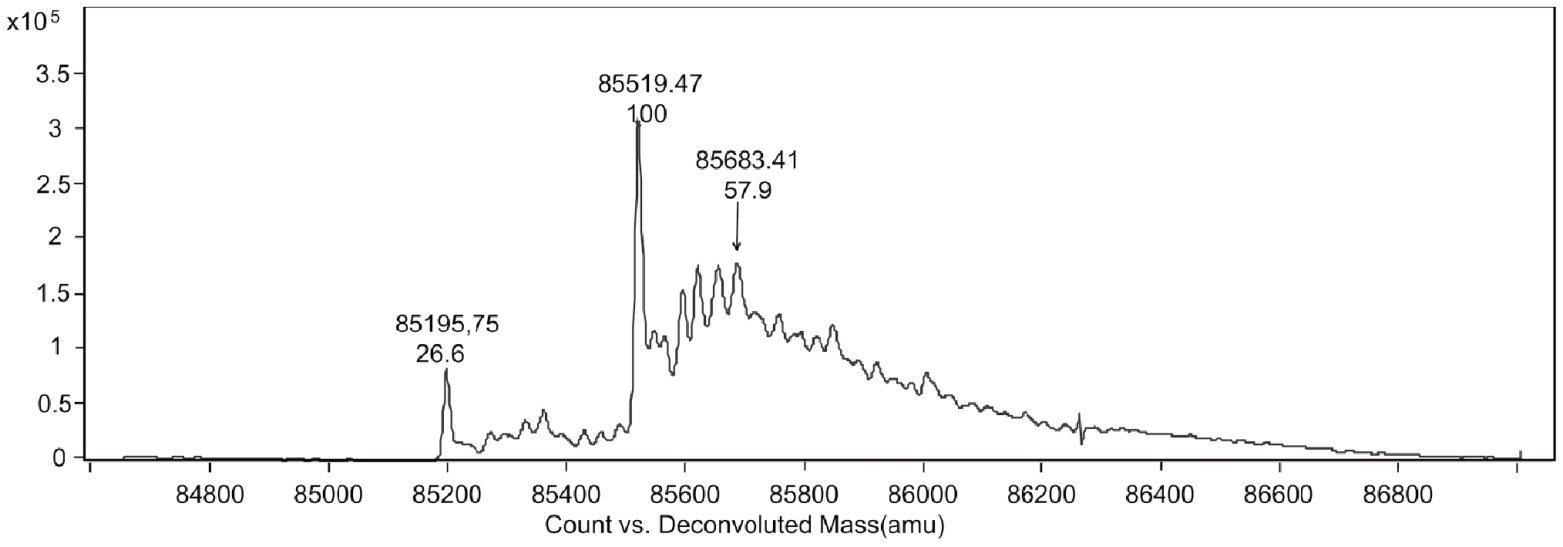
Deconvoluted mass spectra of HMG. Liquid chromatography/mass spectrometry (LC/MS) was used to explore the galactosylated modifications of HMG.

LC/MS was used in further analysis of glycosylation isomers and other potential posttranslational modifications of HMG. A series of peaks with MW greater than 85,195.75 Da (which is close to the theoretical value of HMG, 85,232.9 Da), were observed in the mass spectrum at 85,357 Da, 85,519 Da, 85,683 Da, and 85,844 Da. Of these, 85,519 Da indicated the greatest abundance (Fig. 7). The possibility of acylation was excluded because its contribution to the increase in MW was only 28 or 42 Da. The increase in MW was most possibly the result of glycosylation.

The MWs of a mannose, mannobiose, manninotriose and mannotetraose molecule are 180.2 Da, 342.3 Da, 604.5 Da, and 666.7 Da, respectively. However, one molecule of water is lost during glycosylation, producing a corresponding MW increase of 162.2 Da, 324.3 Da, 586.5 Da, and 648.7 Da, respectively, when a mannose, mannobiose, manninotriose, or mannotetraose is conjugated to an amino acid residue. This indicates that these peaks at 85357 Da, 85519 Da, 85683 Da, and 85844 Da may correspond to 1-, 2-, 3-, and 4-mannose oligosaccharide chains. The areas of the peaks indicate that most of them may be mannobiose. The results of three batches appeared closely consistent (Table 4).

**Table 4:** Characterization of molecular mass distribution of HMG from LC/MS.

MW of observed peaks^a^	Increment to the theoretical MW	Predicted number of mannose residues
85195.3±6D	0	0
85357.2±6D	162.2D	1
85519.8±6D	324.3D	2
85682.1±6D	586.5D	3
85844.3±6D	648.7D	4

a: A deviation of ± 6D was set.

## Discussion

For a fusion protein, retaining individual moiety in the native conformation is essential. For this reason, the structural and functional characteristics of mG-CSF and HSA moieties in HMG were analyzed using various methods. HMG showed a similar CD pattern with emHmG, suggesting that HSA and mG-CSF moieties retained nearly all of their own conformation after fusion. The binding properties of HSA to warfarin were evaluated after fusion and results suggested that this activity of HSA was hardly affected. The G-CSF receptor binding assay revealed that the kinetic binding constants of HMG were similar to those of mG-CSF (Table 2) although a slight decrease of the association/dissociation rate constants was observed in HMG. This may be due to its large molecular size. An *in vitro* cell proliferation assay indicated that HMG did not bind to its target as effectively as mG-CSF did. This may have been because of the steric hindrance effects of HSA, but it remained significantly more potent than rHSA/G-CSF.

However, intrinsic fluorescence measurement failed to provide an unambiguous result, proving that the conformation remained unchanged. The fluorescence spectrum of HMG shifted to a shorter wavelength and the intensity of fluorescence became dramatically less pronounced than that of emHmG and HSA. This may be attributed to the mechanism underlying this form of analysis. The fluorescence spectrum of a folded protein is a mixture of the fluorescence of three aromatic amino acid residues (tryptophan, tyrosine, and phenylalanine), and tryptophan residues contribute most to the emission. mG-CSF alone emits only weak fluorescence, although it contains two Trp in its chain. This may be because they are not sufficiently exposed on the protein's surface. When fused to albumin, mG-CSF may partially block the emission of Trp in albumin, causing the intrinsic fluorescence emissions of HMG to be weaker than those of HSA. Instead, mG-CSF and HSA existed independently in emHmG. They may not interfere with each other, causing emHmG to exhibit a fluorescence pattern similar to that of albumin alone.

Heterologous proteins expressed by *P. pastoris* can be glycosylated on the Asn site of the Asn-Xaa-Thr/Ser consensus sequence or on Ser/Thr hydroxyl groups to provide N-linked or O-linked saccharides, respectively [15]. The N-linked oligosaccharide is commonly a core structure of Man8GlcNAc2, probably originating from a dolichol-linked Glc3Man9GlcNAc2, which elongated on the 1 and 3 arms with a chain of a-1,6-linked mannose units. The O-linked saccharides are generally short (<5 residues) and contain only a1,2-linked mannose units [16]. These kinds of glycosylation patterns of recombinant proteins are generally different from their human-derived counterparts and are frequently responsible for the hyper-antigenic nature and unpredictable pharmacokinetic profiles of these proteins. For example, the high-mannosylated proteins are thought to be linked to mannose binding endocytic receptors in antigen-presenting cells, such as dendritic cells and macrophages [17].

The glycosylation situations of *Pichia*-derived HMG require study in further depth. Fortunately, HMG does not contain any N-glycosylation site, because it lacks the Asn-X-Ser/Thr sequence. It has been reported that *Pichia*-derived rhG-CSF is partially glycosylated with mannose, usually at Thr133 [12], [13]. In the current study, combinational methods were used in a glycosylation assay of HMG. PAS staining demonstrated that HMG may be glycosylated and T718 was considered the most likely site of glycosylation because mHMG. T718→A718 displayed a negative result under PAS staining. In addition, ESI-TOF analysis indicated that HMG may be modified with 1-, 2-, 3-, and 4-mannose (Fig. 7 and Table 4). Mannobiose was the most common of these. This result is supported by phenol-sulfuric acid analysis, which showed HMG to have 0.25% carbohydrate content, equivalent to 1.1–1.28 mannoses attached to each HMG molecule. Oheda *et al.* reported that the sugar chains of CHO-derived G-CSF were a mixture of galactose and galactosamine [18]. It has been thoroughly proven that deglycosylated G-CSF is in effect a mammalian-derived rhG-CSF which has a glycosylation pattern similar to that of native G-CSF. Although glycosylation occurs at the same site as CHO-derived rhG-CSF, the glycan in HMG is a short mannose chain, not galactose or galactosamine [18]. Thus, whether the mannose has an impact on the efficiency and pharmacokinetics of HMG has yet to be determined and further studies are needed to evaluate these problems.

There were some peaks in the mass spectrometric pattern that could not be explained theoretically, but they were not considered a result of phosphorylation. This was because the isoelectric point analysis of HMG showed a single band. We speculated that these peaks may be the results of other modifications, such as oxidization or nitration. Of course, further studies are needed to explore the exact modifications.

In conclusion, a novel HMG fusion protein was here constructed by genetically fusing a mutated human G-CSF to the C-terminus of HSA. The results demonstrated that both mG-CSF and HSA in the fusion protein nearly remained their native structures and the capability of binding to G-CSF receptor and warfarin, respectively. The novel HMG fusion protein exhibited more potent bioactivity than rHSA/G-CSF, which indicated that it may be useful as a therapeutic agent. A glycosylation assay revealed that HMG expressed in *P. pastoris* was glycosylated with mannose at Thr718. However, further studies are needed to explore the possible effect of this glycosylation on the efficiency and pharmacokinetics of HMG.
